# Machine learning models for early prediction of potassium lowering effectiveness and adverse events in patients with hyperkalemia

**DOI:** 10.1038/s41598-024-51468-y

**Published:** 2024-01-06

**Authors:** Wei Huang, Jian-Yong Zhu, Cong-Ying Song, Yuan-Qiang Lu

**Affiliations:** 1 Department of Emergency Medicine, The First Affiliated Hospital, School of Medicine, Zhejiang University, 79 Qingchun Road, Hangzhou, 310003 Zhejiang People’s Republic of China; 2 Key Laboratory for Diagnosis and Treatment of Aging and Physic-Chemical Injury Diseases of Zhejiang Province, Hangzhou, 310003 Zhejiang People’s Republic of China

**Keywords:** Machine learning, Endocrine system and metabolic diseases

## Abstract

The aim of this study was to develop a model for early prediction of adverse events and treatment effectiveness in patients with hyperkalemia. We collected clinical data from patients with hyperkalemia in the First Hospital of Zhejiang University School of Medicine between 2015 and 2021. The least absolute shrinkage and selection operator (LASSO) and multivariate logistic regression were used to analyze the predictors on the full dataset. We randomly divided the data into a training group and a validation group, and used LASSO to filter variables in the training set. Six machine learning methods were used to develop the models. The best model was selected based on the area under the curve (AUC). Shapley additive exPlanations (SHAP) values were used to explain the best model. A total of 1074 patients with hyperkalemia were finally enrolled. Diastolic blood pressure (DBP), breathing, oxygen saturation (SPO2), Glasgow coma score (GCS), liver disease, oliguria, blood sodium, international standardized ratio (ISR), and initial blood potassium were the predictors of the occurrence of adverse events; peripheral edema, estimated glomerular filtration rate (eGFR), blood sodium, actual base residual, and initial blood potassium were the predictors of therapeutic effect. Extreme gradient boosting (XGBoost) model achieved the best performance (adverse events: AUC = 0.87; therapeutic effect: AUC = 0.75). A model based on clinical characteristics was developed and validated with good performance.

## Introduction

Hyperkalemia, defined as serum potassium (K +) > 5.5 mmol/L, is a common electrolyte disorder that can disrupt cell membrane potential and action potential transmission^1^ resulting in potentially life-threatening arrhythmias, and finally is associated with a variety of poor prognoses^2^. The rise in extracellular potassium ions decreases the resting membrane potential, causing nerve, heart, and muscle tissue to depolarize more readily^3^. According to serum potassium levels, hyperkalemia can be classified into three grades: mild (5.5–6.5 mmol/L), moderate (6.5–7.5 mmol/L), and severe (> 7.5 mmol/L)^4^. However, the severity of a patient's clinical presentation not only depends on the serum potassium level but also on the speed of onset, the presence of concomitant electrolyte abnormalities, drug therapy, and other co-morbidities^5^.

Mild hyperkalemia can present with symptoms such as weakness and chest tightness. Severe hyperkalemia is a medical emergency that can trigger fatal arrhythmias such as ventricular fibrillation, cardiac arrest, sudden cardiac death, and other adverse events^6,7^, ultimately leading to significant mortality^8^. According to some studies, potassium itself can cause death, and potassium abnormalities can help identify patients who are sicker, at higher risk of death, or who have heart disease and are physiologically or genetically “unable” to maintain potassium homeostasis^9^. Because of the potentially fatal symptoms that can occur with hyperkalemia, patients often require prompt and aggressive care in the emergency department^6^. In patients admitted to the emergency department, hyperkalemia has been described as an independent predictive feature of death^10^. Physicians need to continuously monitor cardiac function using electrocardiograph in patients with moderate to severe hyperkalemia^7^ and to perform the laboratory assessment of serum potassium levels^11^. Risk factors for hyperkalemia include chronic kidney disease^12^, acute kidney injury (AKI), diabetes, adrenal disease, myocardial dysfunction^13^, and certain medications^4,14^. Hyperkalemia requires dose reduction or even discontinuation of treatment with RAASi1^4,15^ at the expense of long-term cardiac and renal benefits, which is one of the major barriers to controlling disease progression. The estimated risk ratios for increased mortality in hyperkalemia patients compared to non- hyperkalemia patients were reported to be 1.1^16^ to 17.7^17^. It has been shown that serum potassium levels are associated with a U-shaped risk of death, and that low potassium levels also increase the risk of death, but the higher the level of potassium, the higher the risk of death^18^.

A study based on a large U.S. Medicare and commercial claims database containing 1.7 million medical records between 2007 and 2012 showed that the prevalence of hyperkalemia was 34.6% among patients with chronic kidney disease and 30% among patients with heart failure. Hyperkalemia has been a hot topic of clinical research and is being actively explored both in terms of diagnosis and treatment. Hyperkalemia occurs with adverse events associated with higher plasma [K^+^] values^19,20^. There are no studies on the short-term prognosis and treatment outcomes of patients admitted to hospitals with hyperkalemia. Because of the significant increases in hospitalization rate and subsequent in-hospital mortality in hyperkalemia, reliable predictors of adverse clinical outcomes and treatment outcomes have not been established, and it is important to understand the factors associated with treatment efficacy and adverse events in a timely manner^8^. Therefore, we designed this study using a machine learning (ML) algorithm to analyze the clinical data of patients admitted with hyperkalemia in order to develop a model to predict the adverse events and treatment efficacy in patients admitted with hyperkalemia and to screen patients for priority attention.

## Methods

### Data source

Data for this study were obtained from clinical data of patients admitted with hyperkalemia to the emergency department of the First Hospital of Zhejiang University School of Medicine (Hangzhou, China) from January 2015 to December 2021. We collected the detailed basic information, vital sign measurements, diagnostic information, laboratory information, and treatment information. The study was approved by the Clinical Research Ethics Committee of the First Affiliated Hospital, Zhejiang University School of Medicine. Because this study was a retrospective design, written informed consent was waived with the approval of the Clinical Research Ethics Committee of the First Affiliated Hospital, Zhejiang University School of Medicine. This study was performed in line with the principles of the Declaration of Helsinki. Approval was granted by the Clinical Research Ethics Committee of the First Affiliated Hospital, Zhejiang University School of Medicine (No. 2022971).

### Participants

Inclusion criteria: admission diagnosis of hyperkalemia (serum potassium > 5.5 mmol/L); age ≥ 18 years. Exclusion criteria: age < 18 years; pregnant women; laboratory tests suggesting serum potassium < 5.5 mmol /L; incomplete clinical data or missing data on blood potassium; patients with blood potassium > 10.0 mmol/L on any occasion. A total of 1074 patients with hyperkalemia were finally included.

### Research variables

We collected data based on the association of the variables with the outcomes, and then eliminated variables with a missing rate > 28%, and finally selected 52 candidate variables. These variables were recorded for the first time after admission. They included demographic variables, comorbidities, vital signs, laboratory findings, oliguria and Glasgow coma score. Demographic variables included age, gender, smoking, and alcohol consumption. Co-morbidities include hypertension, peripheral edema, diabetes mellitus, heart failure, chronic liver disease, tumors, chronic kidney disease, history of hyperkalemia, diabetic nephropathy, and acute gastrointestinal bleeding. Vital signs included heart rate, systolic blood pressure, diastolic blood pressure, mean arterial pressure, respiratory rate, body temperature, oxygen saturation (SpO_2_), and fraction of inspiration oxygen (FiO_2_). Among the laboratory results, we selected the following variables: white blood cell count, red blood cell count, hemoglobin, red blood cell pressure, platelet count, glutamic aminotransferase, serum creatinine (SCr), estimated glomerular filtration rate (eGFR), urea, uric acid, Initial and last blood potassium, sodium, chloride, total calcium, inorganic phosphorus, International standardized ratio(ISR), fibrinogen (Fib), activated partial thromboplastin time (APTT), prothrombin time (PT), thrombin time (TT), pH, partial pressure of carbon dioxide (pCO_2_), partial pressure of oxygen (pO_2_), bicarbonate concentration, base excess (BE), lactate dehydrogenase (LDH), hydroxybutyrate dehydrogenase (HBDH), and creatine kinase-MB (CKMB). Treatment was considered effective if blood K +  ≤ 5.5 mmol/L checked after the last treatment. Effective and ineffective treatment groups were divided according to the last blood potassium of hospitalization. Admission adverse events included: admission to ICU, death, respiratory and cardiac arrest.

### Study design

Influential factors were analyzed on the full dataset using the least absolute shrinkage and selection operator (LASSO) and multivariate logistic regression. In this retrospective cohort study, model development, validation, interpretation, and application were performed sequentially. We divided the overall random data into two parts, where 70% is used as training data and 30% as validation data. Use LASSO to filter variables in the training set. For the prediction of adverse events, the data in the training set were balanced using the SMOTE algorithm. Six ML models—XGBoost, logistic regression (LR), random forest (RF), support vector machine (SVM), k-nearest neighbor (KNN), and decision tree (DT)—were used to build predictive models. In order to improve the fairness and reliability of the comparison between models, this study used tenfold cross-validation to initially assess the performance. The data was normalized using the MinMaxScaler function in sklearn.preprocessing module before applying KNN, SVM, logistic. First, we found the optimal parameters for each of the six machine learning methods by grid search and fivefold cross-validation in the training set, and then validated them in the test set. Second, the method with the largest area under the curve (AUC) was selected for modeling. In addition, we calculated accuracy, sensitivity, specificity and F1 scores. Some algorithms were randomized, resulting in different results each time they were executed. Each algorithm was run 1200 times to reduce the possible bias introduced. Decision curves were used to assess clinical benefits. Finally, we used the SHAP method to illustrate our final model. SHAP summary plots were used to illustrate the impact of features attributed to the model. SHAP force diagrams were used to visualize the impact of key features on the final model for individual patients.

### Statistical analysis

Categorical data were expressed as frequencies and percentages, and differences between groups were compared by chi-square test or Fisher's test. Continuous variables that did not conform to a normal distribution, denoted as median and interquartile range (IQR), were compared between the two groups using the Wilcoxon rank sum test. Continuous variables conformed to a normal distribution expressed as mean and standard deviation, and t-tests were used when comparing the two groups. Missing data were used the “mice” package in R to impute. Correlation between variables were analyzed using spearman. All analyses were performed using Python (v. 3.8.3) and R (v. 4.2.1, R statistical computing base). Two-tailed *P* values < 0.05 indicated statistical significance.

## Results

### Patient characteristics

A total of 1074 adult patients with a diagnosis of hyperkalemia were included in the final cohort of this study. The data set was randomly divided into 2 parts:70% (adverse events: n = 1372; therapeutic effect: n = 751) of the data were used for model training and 30% (adverse events: n = 323; therapeutic effect: n = 323) of the data were used for model validation (Supplementary Table 2; Supplementary Table 3). The group with adverse events was significantly older than the group without adverse events (*P* < 0.05) (Supplementary Table 1). The incidence of unsuccessful treatment was 35.553% (267/751) in the training data set and 35.604% (115/323) in the validation data set (Supplementary Table 3). Table 1 and supplementary Table 1 compare all candidate variables between the two patient groups.

**Table 1:** Demographic and clinical characteristics at baseline.

		Last blood potassium ≤ 5.5 (n = 692)	Last blood potassium > 5.5 (n = 382)	*P* value
Age, year		64 (53, 77)	66 (55.00, 78.00)	0.127
Gender (male), n (%)	1	632 (91.329)	57 (14.921)	0.625
Temperature, ℃		36.7 (36.3, 37.0)	36.5 (36.1, 36.98)	0.002
Heart rate, bpm		84 (74.0, 98.0)	85 (72.00, 102.00)	0.927
SBP, mmHg		134.0 (114.0, 155.0)	136.5 (114.2, 159.00)	0.234
DBP, mmHg		73.0 (60.0, 87.0)	75 (62.25, 88.00)	0.445
MAP, mmHg		94.17 (80.67, 107.42)	96 (82.42, 109.58)	0.205
breathing		20 (19.0, 20.0)	20.00 (20.00, 22.00)	0.003
SpO_2_, %		99 (97.75, 100.0)	98.00 (96.00, 100.00)	0.005
FiO_2_, mmHg		21.0 (21.0, 21.0)	21.00 (21.00, 21.00)	0.713
GCS		15.0 (15.00, 15.00)	15.00 (15.00, 15.00)	0.212
Edema, n (%)	1	169 (24.422)	130 (34.031)	0.001
Smoking, n (%)	1	174 (25.145)	95 (24.869)	0.979
Drinking, n (%)	1	144 (20.809)	58 (15.183)	0.029
HTN, n (%)	1	458 (66.185)	260 (68.063)	0.577
DM, n (%)	1	192 (27.746)	120 (31.414)	0.231
Heart failure, n (%)	1	100 (14.451)	59 (15.445)	0.727
Liver disease, n (%)	1	79 (11.416)	53 (13.874)	0.281
Tumors, n (%)	1	123 (17.775)	76 (19.895)	0.439
Chronic kidney disease, n (%)	1	414 (59.827)	249 (65.183)	0.096
Diabetic Nephropathy, n (%)	1	50 (7.225)	33 (8.639)	0.477
Oliguria, n (%)	1	111 (16.040)	98 (25.654)	< 0.001
High potassium history, n (%)	1	46 (6.647)	30 (7.853)	0.540
Acute gastrointestinal bleeding, n (%)	1	62 (8.960)	36 (9.424)	0.887
Leukocyte count, 10^9/L		8.10 (6.00, 12)	8.9 (6.3, 13)	0.019
Erythrocyte count, 10^12/L		3.405 (2.678, 4.013)	3.18 (2.56, 3.83)	0.003
Hemoglobin, g/L		102 (81.0, 121.0)	95.00 (77.00, 114.00)	0.001
Erythrocyte pressure product, n (%)		31.3 (25.4, 37.12)	29.8 (23.90, 35.30)	0.003
Platelet count, 10^9/L		187 (136.0, 255.2)	180 (126.5, 248.80)	0.293
Glutathione transaminase, U/L		20.00 (14, 37.00)	21 (14.00, 42.00)	0.66
Creatinine, μmol /L		287.5 (163, 623.5)	458.5 (234.5, 791.8)	< 0.001
eGFR		17.04 (17.04, 33.66)	9.835 (5.612, 22)	< 0.001
Urea, mg/dL		20.35 (14.07, 29.61)	24.51 (18.23, 35.22)	< 0.001
Uric acid, μmol/L		448.5 (357.8, 557)	467 (364.0, 605.5)	0.056
Sodium, mmol/L		138.0 (135, 141)	137.0 (133.0, 140.0)	0.001
Chlorine, mmol/L		104 (98, 108)	102.00 (96.00, 107.00)	0.002
Total calcium, mmol/L		2.17 (2.0, 2.32)	2.12 (1.972, 2.280)	0.032
Inorganic phosphorus, mmol/L		1.56 (1.26, 2.02)	1.74 (1.37, 2.305)	< 0.001
ISR		1.08 (1, 1.25)	1.08 (1, 1.288)	0.477
Fib, g/L		3.58 (2.777, 4.692)	3.74 (2.76, 4.888)	0.466
APTT, second		29.8 (26.57, 35.6)	30.2 (26.1, 36.17)	0.534
PT, second		12.5 (11.7, 14.43)	12.5 (11.7, 14.6)	0.546
TT, second		17.7 (16.8, 18.9)	17.7 (16.82, 18.9)	0.689
pH		7.38 (7.316, 7.42)	7.355 (7.29, 7.4)	< 0.001
pCO_2_, mmHg		34.8 (29.48, 39.6)	33.6 (28.5, 39.98)	0.133
pO_2_, mmHg		95.55 (69.85, 119)	100.5 (69.45, 129.00)	0.246
Bicarbonate concentration, mmol/L		20.05 (16.6, 23.5)	18.5 (14.6, 22.07)	< 0.001
Base excess, mmol/L		− 4.1 (− 8.025, − 0.7)	− 6.2 (− 10.2, − 2.3)	< 0.001
LDH, U/L		253 (200, 340)	260.5 (216.0, 373.8)	< 0.020
HBDH, U/L		214 (165, 279)	224 (180.2, 320)	0.004
CKMB, U/L		19 (13.00, 28.00)	19.00 (14.00, 32.00)	0.143
Initial potassium, mmol/L		6.34 (6.14, 6.66)	6.575 (6.3, 6.96)	< 0.001

*SBP* systolic blood pressure, *DBP* diastolic blood pressure, *MAP* mean arterial pressure, *SpO*_*2*_ oxygen saturation, *FiO*_*2*_ fraction of inspiration oxygen, *GCS* Glasgow coma score, *HTN* hypertension, *DM* diabetes mellitus, *eGFR* estimated glomerular filtration rate, *ISR* international standardized ratio, *Fib* fibrinogen, *APTT* activated partial thromboplastin time, *PT* prothrombin time, *TT* thrombin time, *pCO*_*2*_ partial pressure of carbon dioxide, *pO*_*2*_ partial pressure of oxygen, *LDH* lactate dehydrogenase, *HBDH* hydroxybutyrate dehydrogenase, *CK-MB* creatine kinase-MB, 1 yes.

### Risk characterization factors for treatment effects and adverse events

The LASSO compresses variable coefficients to prevent overfitting and to address severe covariances. LASSO regression analysis was performed on the full dataset to screen variables (Supplementary Fig. 1; Supplementary Fig. 2). The results showed that 13 variables were screened for adverse events: DBP, breathing, SpO_2_, GCS, liver disease, oliguria, urea, uric acid, sodium, ISR, PH, BE, and initial blood potassium. The treatment effects were screened for 7 variables: peripheral edema, oliguria, eGFR, urea, sodium, BE, and initial blood potassium. The correlations between the variables were all low (Supplementary Fig. 3). To further control for the effects of confounding factors, multivariate logistic regression analysis was performed. Finally, only peripheral edema, eGFR, sodium, base excess, and Initial blood potassium were identified as influences on treatment effect (Table 2). Only DBP, breathing, SpO_2_, GCS, liver disease, oliguria, sodium, ISR, and initial potassium were identified as factors affecting adverse events (Supplementary Table 4).

**Table2:** Multivariate logistic regression analysis of therapeutic effect.

Variables	R	SE	Z	*P*	OR	OR (95%CI)
Peripheral edema						
0					1	
1	0.362	0.150	2.418	0.016	1.436	1.071–1.925
eGFR	− 0.014	0.004	− 3.265	0.001	0.986	0.978–0.994
Sodium	− 0.042	0.011	− 3.703	< 0.001	0.959	0.938–0.980
Base excess	− 0.027	0.011	− 2.396	0.017	0.973	0.952–0.995
Initial potassium	0.982	0.139	7.088	< 0.001	2.671	2.035–3.504

*R* regression coefficient, *SE* standard error, *OR* odds ratio, *CI* confidence interval, *eGFR* estimated glomerular filtration rate, 0 no, 1 yes.

LASSO regression analysis was performed on the training set to screen the variables. (Supplementary Fig. 4; Supplementary Fig. 5). The results showed that 15 variables were screened for adverse events: DBP, breathing, SpO_2_, GCS, liver disease, oliguria, Fib, uric acid, sodium, ISR, PH, BE, glutathione transaminase, FiO_2_, and initial blood potassium. The treatment effects were screened for 7 variables: peripheral edema, oliguria, eGFR, urea, sodium, BE, Hemoglobin, and initial blood potassium. The correlations between the variables were all low (Supplementary Fig. 6).

### Model building and evaluation

**Figure 1:**
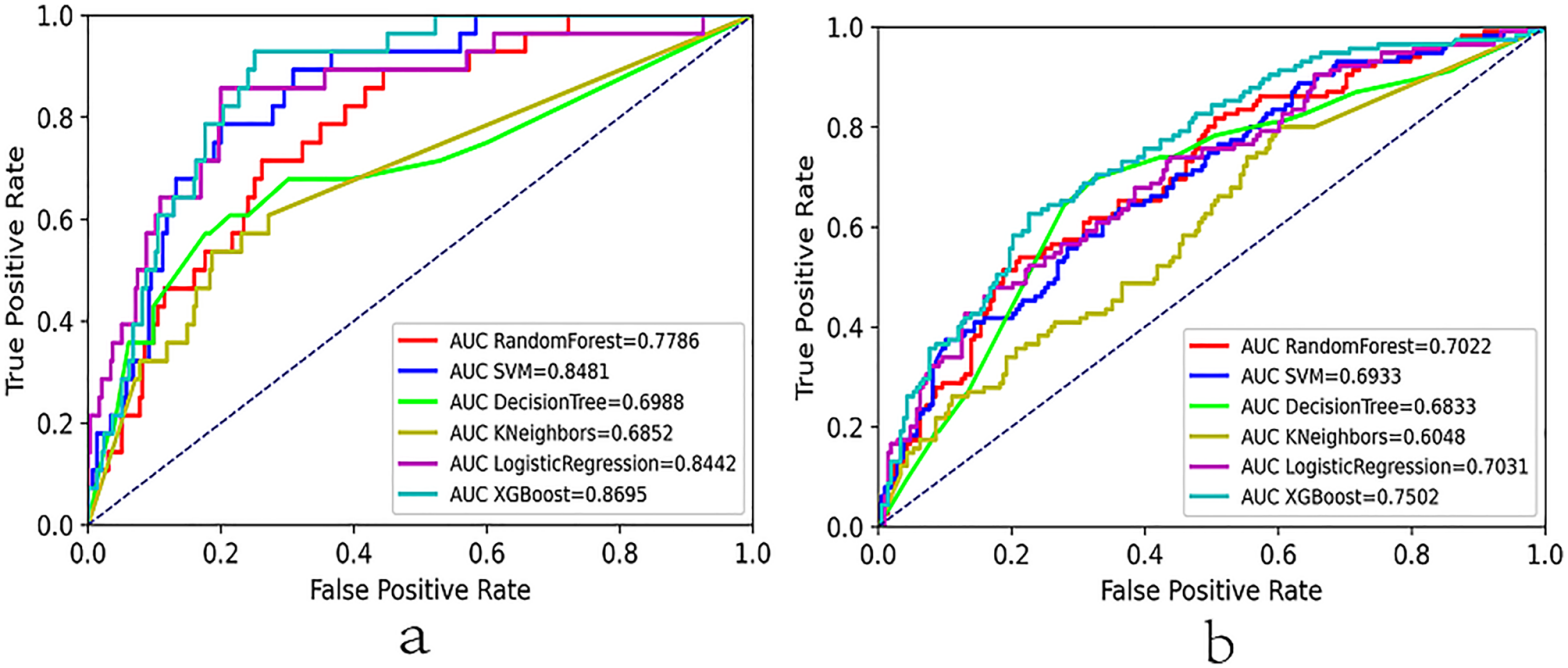
Evaluation of the six machine learning algorithms based on the AUC of the ROC curve. AUC, area under the curve; ROC, receiver operating characteristic; SVM, support vector machine; XGBoost, extreme gradient boosting; (**a**) adverse events; (**b**) therapeutic effect.

**Figure 2:**
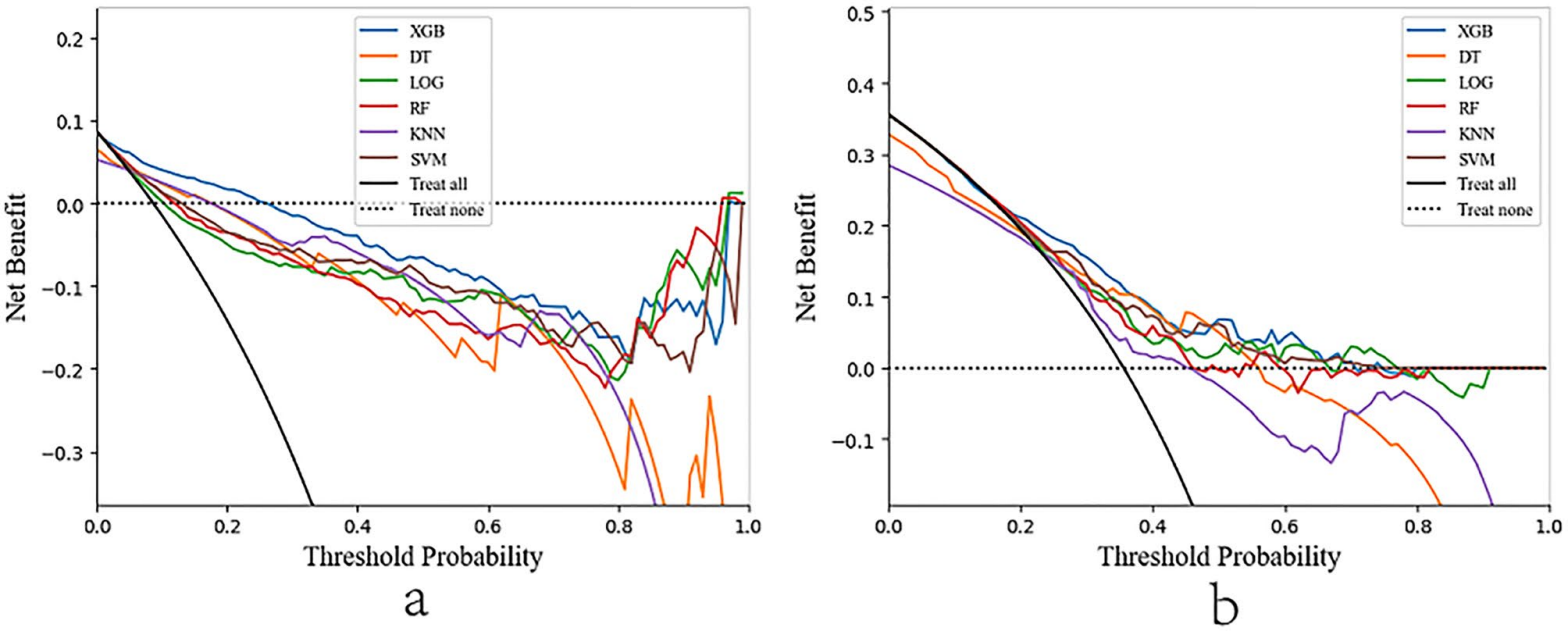
Decision curve analysis for 6 models. AUC, area under the curve; XGBoost, extreme gradient boosting; LR, logistic regression; KNN, k-nearest neighbor; DT, decision tree; SVM, support vector machine; RF, random forest; (**a**) adverse events; (**b**) therapeutic effect.

Tenfold cross-validation was performed for performance evaluation. Using AUC values as the evaluation metrics and plotting box plots to initially see the distribution of predictive performance, the results showed the best performance of the XGBoost model (Supplementary Fig. 7). The LASSO-screened variables were used to build models in the training set and to predict in the test set. In Table 3 and Supplementary Table 5, we summarized the performance of the six models in terms of AUC, accuracy, sensitivity, specificity and F1 scores. In the prediction of adverse events, compared with other ML models (AUC: RF 0.779, LR 0.844, SVM 0.848, KNN 0.685, DT 0.699; accuracy: RF 0.780, LR 0.796, SVM 0.827, KNN 0.814, DT 0.770, XGBoost 0.848) (Supplementary Table 5), the XGBoost model had the best model fit performance with an AUC of 0.870 (a of Fig. 1) and a sensitivity of 0.643 in the validation cohort. The DCA measures net benefit at different threshold probabilities. The black line in Fig. 2 indicates that all patients were assumed to receive the intervention, while the dashed line indicates that all patients did not receive the intervention. The threshold probabilities range from 0.016 to 0.258, and the XGBoost model outperforms the other models in terms of net benefit (a of Fig. 2).

**Table 3:** Performances of the six machine learning models for predicting therapeutic effect.

	XGBoost	LR	KNN	DT	SVM	RF
AUC	0.750	0.703	0.605	0.683	0.693	0.702
Accuracy	0.712	0.666	0.619	0.694	0.613	0.641
F1 scores	0.611	0.535	0.428	0.599	0.549	0.554
Sensitivity	0.589	0.539	0.400	0.644	0.661	0.626
Specificity	0.635	0.530	0.460	0.561	0.469	0.497

*AUC* area under the curve, *XGBoost* extreme gradient boosting, *LR* logistic regression, *KNN* k-nearest neighbor, *DT* decision tree, *SVM* support vector machine, *RF* random forest.

In predicting treatment effects, compared with other ML models (AUC: RF 0.702, LR 0.703, SVM 0.693, KNN 0.605, and DT 0.683; accuracy: RF 0.641, LR 0.666, SVM 0.613, KNN 0.619, and DT 0.694), in the validation cohort, the XGBoost model has the best model fit performance with an AUC of 0.750 and an accuracy of 0.712 (Table 3). However, the SVM model has the highest sensitivity (0.661). For threshold probabilities from 0.183 to 0.435 (or 0.488–0.685), the XGBoost model outperforms the other models in net benefit (b of Fig. 2). After considering several performance metrics together, we chose XGBoost to construct the final model. Random seeds were removed and the mean AUC and standard deviation were obtained by running each algorithm 1200 times (Supplementary Table 6; Supplementary Table 7). It could be seen that the final choice of XGBoost model was also stable.

### Model explanation

**Figure 3:**
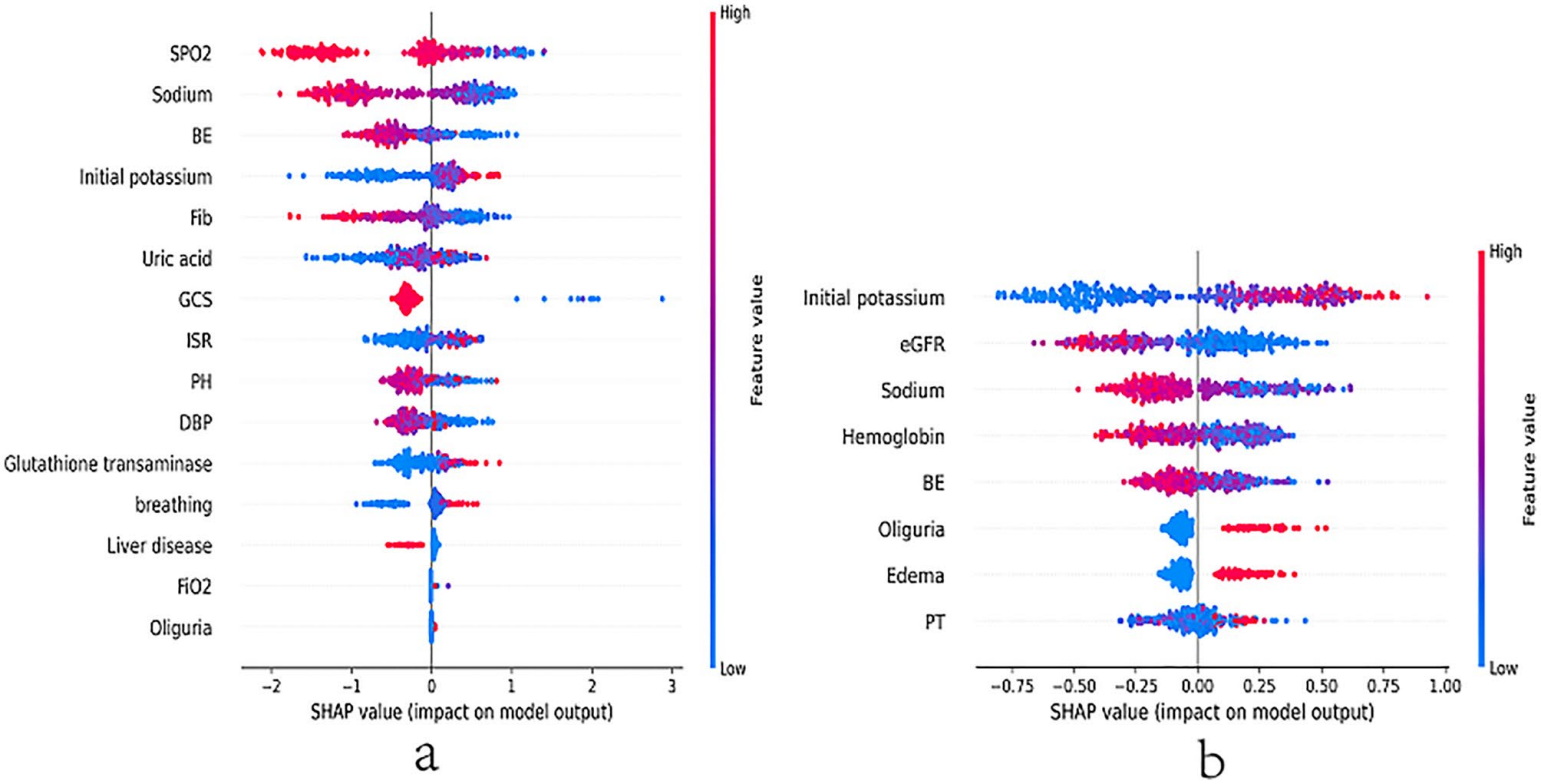
SHAP summary plot of the features of the XGBoost model. The higher the SHAP value of a feature, the higher the probability of an adverse event. A point is created for each feature attributed value of the model for each patient, so that one point is assigned to a patient on a straight line for each feature. The dots are colored according to the patient's feature values and accumulated vertically to describe the density. Red represents higher feature values; blue represents lower feature values. SHAP, SHapley Additive exPlanations; (**a**) adverse events; (**b**) therapeutic effect; DBP, diastolic blood pressure; SpO_2_, oxygen saturation; GCS, Glasgow coma score; ISR, international standardized ratio; eGFR, estimated glomerular filtration rate; BE, base excess; FiO_2,_ fraction of inspiration oxygen; Fib, fibrinogen; PT, prothrombin time.

The SHAP algorithm was used to derive the importance of each predictive feature on the prediction results of the XGBoost model. The feature importance plot lists the relatively significant features by descending order (Supplementary Fig. 8). SHAP feature density scatter plots showed that SpO_2_ had the strongest predictive value, followed by sodium, BE and initial potassium. In addition, to detect positive and negative relationships between features and target outcomes, SHAP values were applied to reveal risk factors for the occurrence of adverse events in hyperkalemia (a of Fig. 3). Figure 3 showed the distribution of all individuals on each variable, where the horizontal coordinates measured the size of the variable as it got larger to the right. The effects of features on the XGBoost model (positive or negative) are shown in Fig. 3^21^, red represents an increased risk of death and blue represents a decreased risk of death. SHAP feature density scatter plots. It can be seen that the presence of uric acid had a positive effect and drives the prediction of an adverse event, while an increase in SPO2 had a negative effect and drives the prediction of no adverse event.

SHAP feature density scatter plots showed that initial potassium had the strongest predictive value, followed by eGFR, sodium and hemoglobin (b of Fig. 3). It can be seen that the presence of initial potassium had a positive effect and drives the prediction of an unsuccessful treatment, while an increase in eGFR had a negative effect and drives the prediction of successful treatment.

### Model application

**Figure 4:**
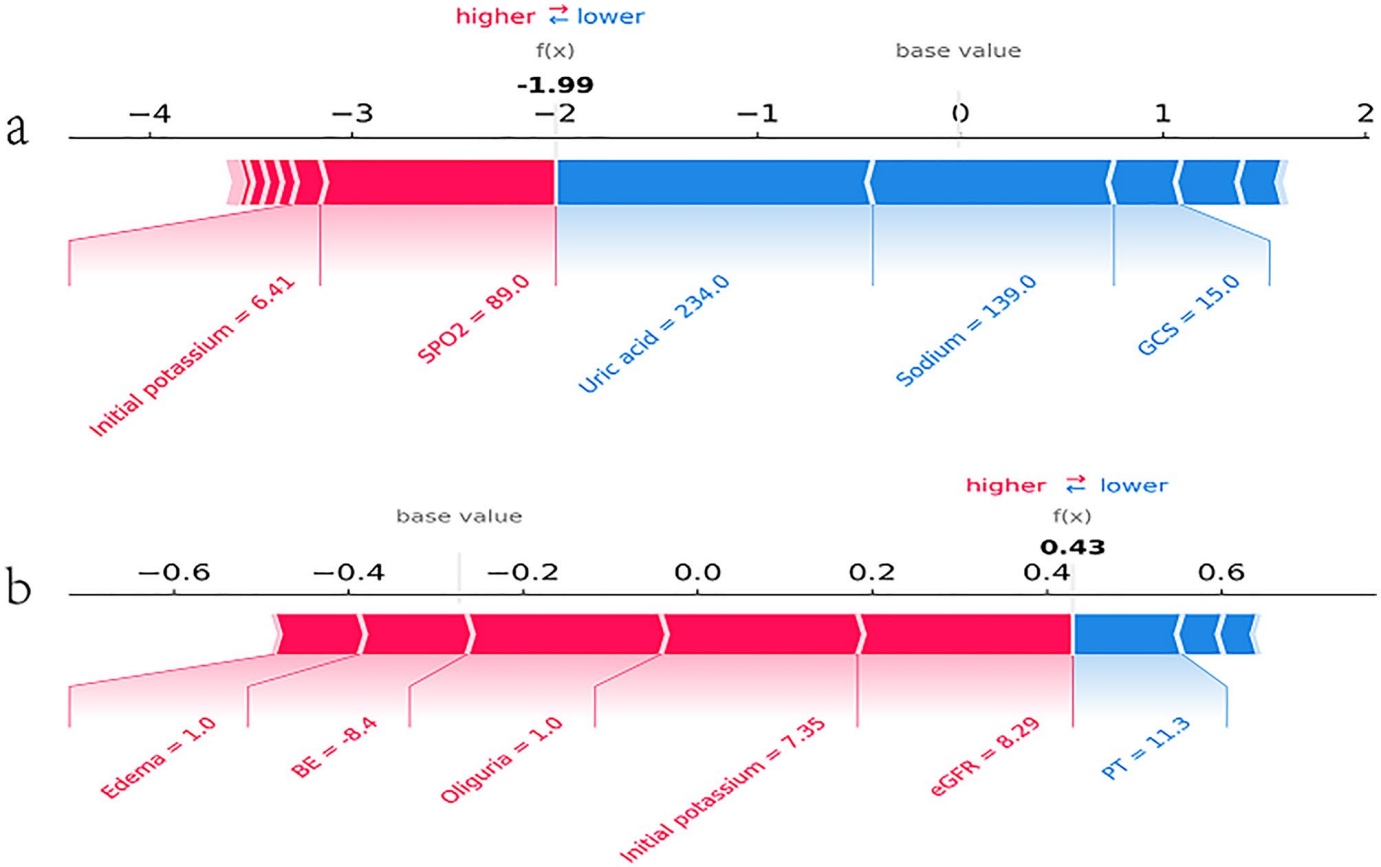
Force plot of model prediction results explained with a random sample. eGFR, estimated glomerular filtration rate; BE, base excess; SpO_2_, oxygen saturation; GCS, Glasgow coma score; PT, prothrombin time; (**a**) adverse events; (**b**) therapeutic effect;

Figure 4 show the individual force maps for randomly selected patients without adverse events (a) and unsuccessful treatment (b). This patient’s SHAP value indicates the predictive variables of relevance for the individual patient and the contribution of each variable to the prediction of target event. The number on the X-axis is the SHAP value, and the values for each feature of the sample are shown below the horizontal line. Red features indicate an increased risk of target event, and blue features indicate a decreased risk of target event^22^. The length of the arrow is proportional to the SHAP value, the longer the arrow, the greater the prediction effect^23^. The contribution of some variables is too low to be shown in the figure, and only the more contributing variables are shown in Fig. 4. SHAP value for target event in patient A was − 1.99, and the actual patient survived after admission. SHAP value for target event in patient B was 0.43, and the actual patient's last potassium was greater than 5.5.

## Discussion

Previously, no studies were predicting adverse events and therapeutic effect in patients with hyperkalemia admitted to the hospital, so this study is open source. The aim of this study was to predict the final potassium-lowering effect in hyperkalemic patients at an early stage, i.e., to screen out patients who are not prone to successful potassium-lowering prior to treatment, and subsequently to focus attention and treatment. Therefore, therapeutic drugs and duration of treatment were not included in this study for analysis. In this retrospective cohort study in emergency medicine, we developed and validated six ML algorithms. The XGBoost model outperformed LR, RF, SVM, KNN, and DT. The interpretation of the XGBoost model using the SHAP method ensured the clinical interpretability of the model, which allowed physicians to better understand the decision process of the model and facilitated the use of the model. In this study, we found that the developed model performs best when the DCA correlation threshold probability is in a certain range. XGBoost has been widely used to predict in-hospital mortality in patients in numerous studies. However, the rate of adverse events in the final cohort of patients with hyperkalemia was only 8.66%. The ROC curve indicated that the XGBoost model was the best, but the accuracy of the adverse event class prediction was 0.643 (sensitivity). Therefore, the XGBoost model might not fully provide decision support for clinicians. In clinical practice, it is necessary to evaluate the benefits of early prediction of adverse events and their additional costs.

The SHAP method shows the contribution of all variables to the model output not only at the macro level through the feature density scatter plots, feature importance SHAP values, but also at the micro level through the individual sample variable impact plots^24^. By using SHAP to interpret the XGBoost model, we identified a number of important variables associated with adverse events and therapeutic effect of hyperkalemia with hospital admission. SHAP specifies whether the effects of variables are positive or negative^25^. In the present study, SpO_2_ and initial potassium were the most important predictive feature. A more serious consequence of hyperkalemia is a decrease in myocardial resting membrane potential, which leads to a slowing of myocardial cell conduction velocity and an increase in repolarization rate^3^. Hyperkalemia has a significant effect on both conduction and automaticity of cardiomyocytes^26^, Both high potassium and low sodium affect the electrophysiological activity of cardiomyocytes, and potassium is necessary for normal cardiomyocyte function including impulse conduction and coordinated myocardial contraction^9,27^. Thus, disturbances in potassium levels predispose to arrhythmias, and the mechanism by which high potassium causes death in patients may be the induction of fatal arrhythmias^28^. Reduced tubular flow due to sodium restriction may lead to hyperkalemia^29^, and disturbances in both serum sodium and potassium are independently associated with poor prognosis^30^. The kidneys are the most important excretory site^31^, and when kidney function is abnormal and excretory function is impaired, it will lead to an increase in uric acid concentration. The decline in glomerular filtration capacity is a direct reflection of progressive kidney damage. Elevated levels of urea indicate an increased risk of AKI^32^. Under normal conditions, the kidneys excrete 90% of the daily potassium intake^12,33,34^, and abnormal kidney function is the most common cause of hyperkalemia^18,35,36^, so for patients with hyperkalemia, we should focus on the patient's kidney function^37^. In addition, patients with peripheral edema or oliguria were also prone to bad outcomes, and these symptoms might also suggest renal dysfunction^38^. Metabolic acidosis occurs in all patients prior to cardiac arrest, which can result in extracellular potassium transfer, when the base excess is too low^5,33,39^. It has been suggested that hyperkalemia can cause renal tubular acidosis and lead to peripheral neuropathy in patients with chronic kidney disease^34^. It has been noted that metabolic acidosis and AKI are independent predictors of mortality in patients hospitalized with hyperkalemia^40^. The GCS score is a level of consciousness score that can clearly indicate deterioration in neurological function; a lower score indicates a worse condition, and the GCS score is often associated with the risk of death. Hyperkalemia increases the risk of adverse events associated with arrhythmias, which can lead to hypotension and myocardial ischemia^39^. In addition, higher ISR indicates a worse prognosis for the patient, which is consistent with the actual clinical significance.

There are some limitations in this study. First, variables with missing values and high rates of missingness were removed from this study, and potentially more important characteristics were not selected for inclusion. Second, all data were from China, and there were many unmeasured confounders, such as race and treatment strategy. Third, our study lacked external validation of independent cohorts from other regions or countries, and the applicability of the developed XGBoost model in clinical practice needs further validation. Fourth, a single-center retrospective study limited our ability to identify causal relationships between variables and outcomes. Therefore, further prospective randomized controlled trials are needed to validate the validity of our model. Finally, only adults were recruited in our study, and the predictive effect of the XGBoost model on the prognosis and therapeutic effect of children with hyperkalemia is unclear. This finding needs to be interpreted with caution, and more evidence is needed to confirm it in the near future.

## Conclusions

For hyperkalemia, we developed the interpretable XGBoost prediction model that performed best in predicting the risk of adverse events and therapeutic effect. In addition, applying interpretable ML can accurately identify risk factors and enhance physician confidence in the prediction model. This will help physicians to identify hyperkalemia patients with a high risk of death so that appropriate treatment measures can be taken promptly.

### Supplementary Information

Supplementary Information.

## Data Availability

De-identified data and associated code can be made available from the corresponding author upon reasonable request. Contact information for the corresponding author is included on the title page.
