# Supplementary material for: Machine learning models for early prediction of potassium lowering effectiveness and adverse events in patients with hyperkalemia
Source: Sci Rep. 2024 Jan 6;14:737. doi: 10.1038/s41598-024-51468-y (PMC10771443; doi:10.1038/s41598-024-51468-y)
Supplement: Supplementary file 1 — Supplementary Information. [file 41598_2024_51468_MOESM1_ESM.pdf]

Supplemental Digital Content

Title: Machine learning models for early prediction of potassium lowering effectiveness and adverse events in patients with hyperkalemia

Authors: Wei Huang, MD; Jian-Yong Zhu, MD; Cong-Ying Song, MD; Yuan-Qiang Lu MD, PhD

Figure and Table Contents

Additional

Supplemental Digital Content Figure 1……………………………..…… Page 2
Supplemental Digital Content Figure 2…………………………..………Page 2
Supplemental Digital Content Figure 3…………………………..………Page 3
Supplemental Digital Content Figure 4…………………………..………Page 3
Supplemental Digital Content Figure 5……………………………..…… Page 4
Supplemental Digital Content Figure 6…………………………..………Page 4
Supplemental Digital Content Figure 7…………………………..………Page 5
Supplemental Digital Content Figure 8…………………………..………Page 5
Supplemental Digital Content Table 1…………………………..………Page 6
Supplemental Digital Content Table 1…………………………..………Page 7
Supplemental Digital Content Table 2…………………………..………Page 8
Supplemental Digital Content Table 3…………………………..………Page 9
Supplemental Digital Content Table 4…………………………..………Page 10
Supplemental Digital Content Table 5…………………………..………Page 10
Supplemental Digital Content Table 6…………………………..………Page 11
Supplemental Digital Content Table 7…………………………..………Page 11

Supplementary Fig. 1 LASSO regression analysis was used on the full dataset to select variables affecting adverse events.

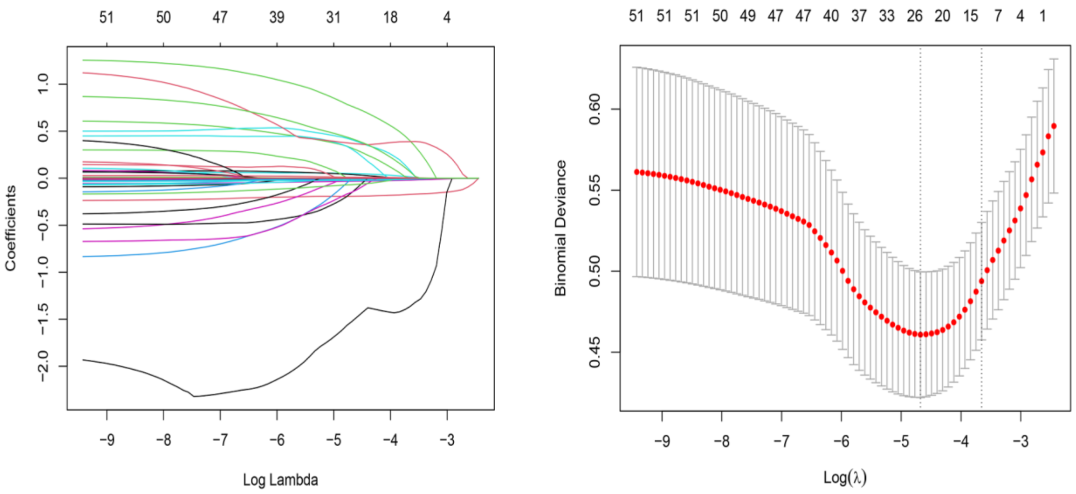

A B

(A) The use of 10-fold cross-validation select values. (B) In the LASSO model, the coefficient profiles of 52 texture features were drawn from the log (λ) sequence. Vertical dotted lines are drawn at the minimum mean square error and the standard error of the minimum distance. In this study, the standard error of the minimum distance was selected.

Supplementary Fig. 2 LASSO regression analysis was used on the full dataset to select variables affecting therapeutic effect.

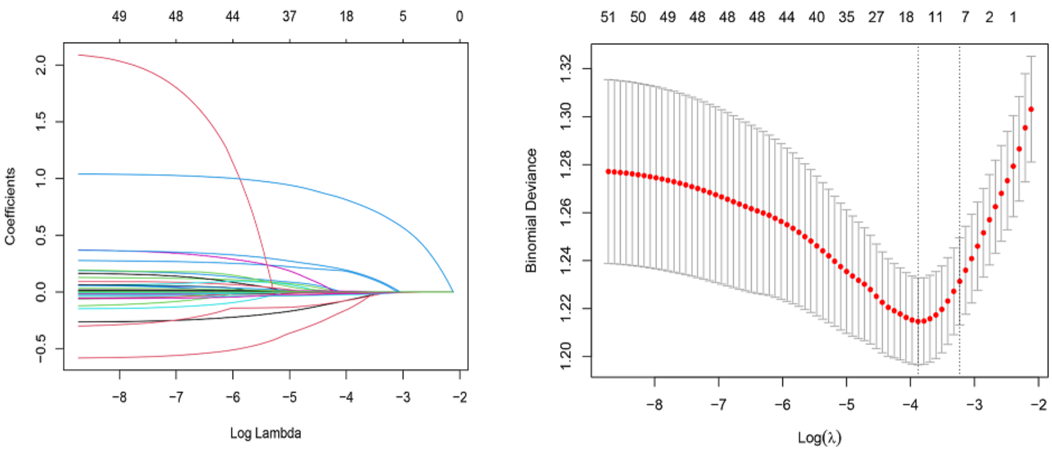

A B

(A) The use of 5-fold cross-validation select values. (B) In the LASSO model, the coefficient profiles of 52 texture features were drawn from the log (λ) sequence. Vertical dotted lines are drawn at the minimum mean square error and the standard error of the minimum distance. In this study, the standard error of the minimum distance was selected.

Supplementary Fig. 3 Correlation analysis.

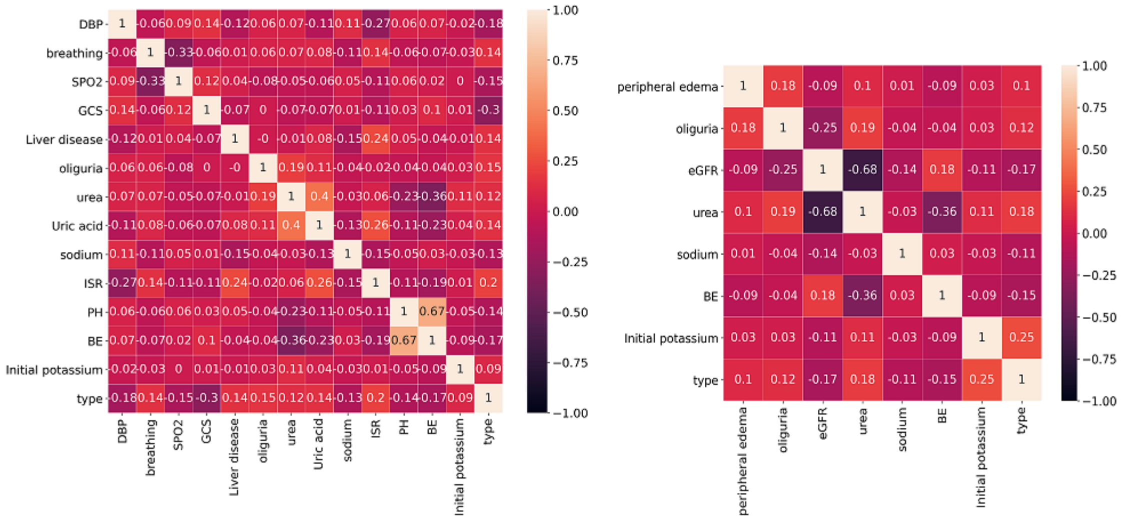

A B

spearman was used for correlation analysis. A, adverse outcomes; B, treatment effectiveness; Abbreviations: DBP, diastolic blood pressure; SpO_2, oxygen saturation; GCS, Glasgow coma score; ISR, International standardized ratio; eGFR, estimated glomerular filtration rate; BE, base excess.

Supplementary Fig. 4 LASSO regression analysis was used on the training dataset to select variables predicting adverse outcomes.

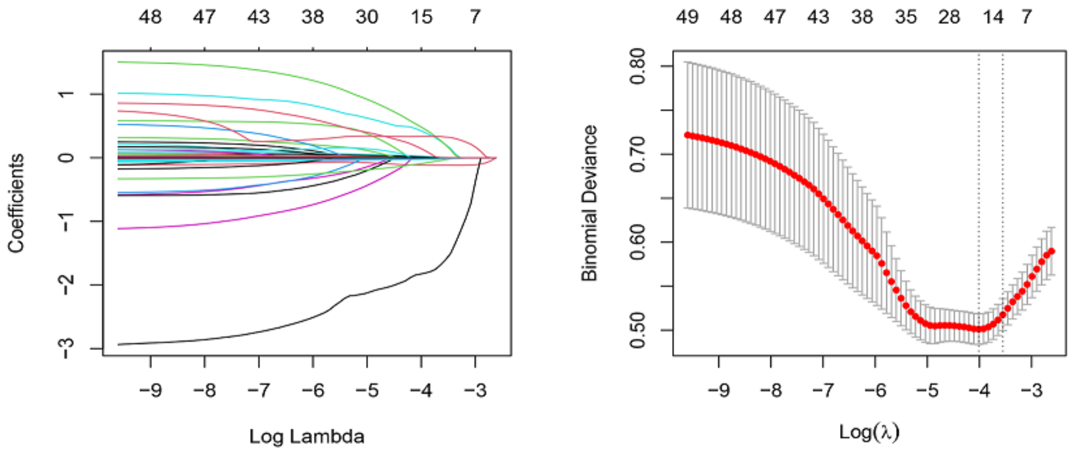

A B

(A) The use of 10-fold cross-validation select values. (B) In the LASSO model, the coefficient profiles of 52 texture features were drawn from the log (λ) sequence. Vertical dotted lines are drawn at the minimum mean square error and the standard error of the minimum distance. The left dashed line was selected to construct the model for this study.

Supplementary Fig. 5 LASSO regression analysis was used on the training dataset to select variables predicting therapeutic effect.

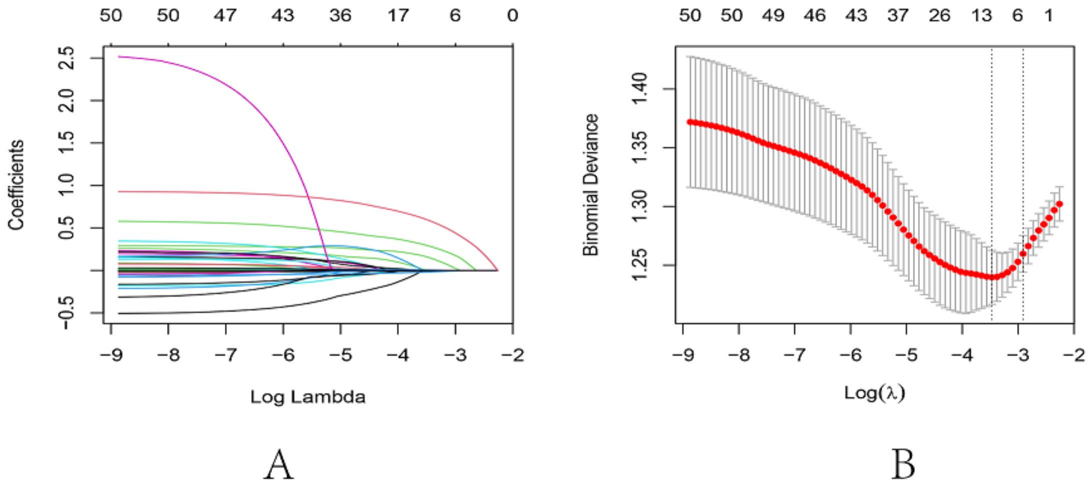

(A) The use of 10-fold cross-validation select values. (B) In the LASSO model, the coefficient profiles of 52 texture features were drawn from the log (λ) sequence. Vertical dotted lines are drawn at the minimum mean square error and the standard error of the minimum distance. The left dashed line was selected to construct the model for this study.

Supplementary Fig. 6 Correlation analysis.

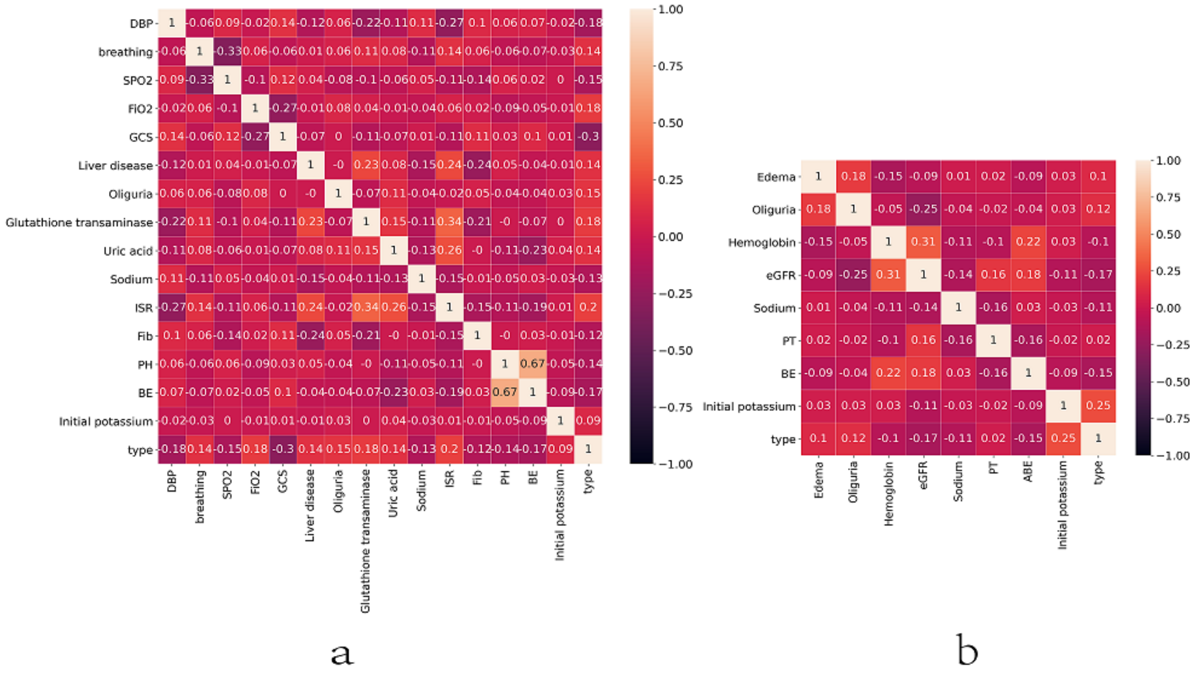

spearman was used for correlation analysis. A, adverse outcomes; B, treatment effectiveness; Abbreviations: DBP, diastolic blood pressure; SpO_2, oxygen saturation; GCS, Glasgow coma score; ISR, International standardized ratio; eGFR, estimated glomerular filtration rate; BE, base excess; FiO_2, fraction of inspiration oxygen; Fib, fibrinogen; PT, prothrombin time.

Supplementary Fig. 7 Comparison of box diagrams. The green triangles represent averages.

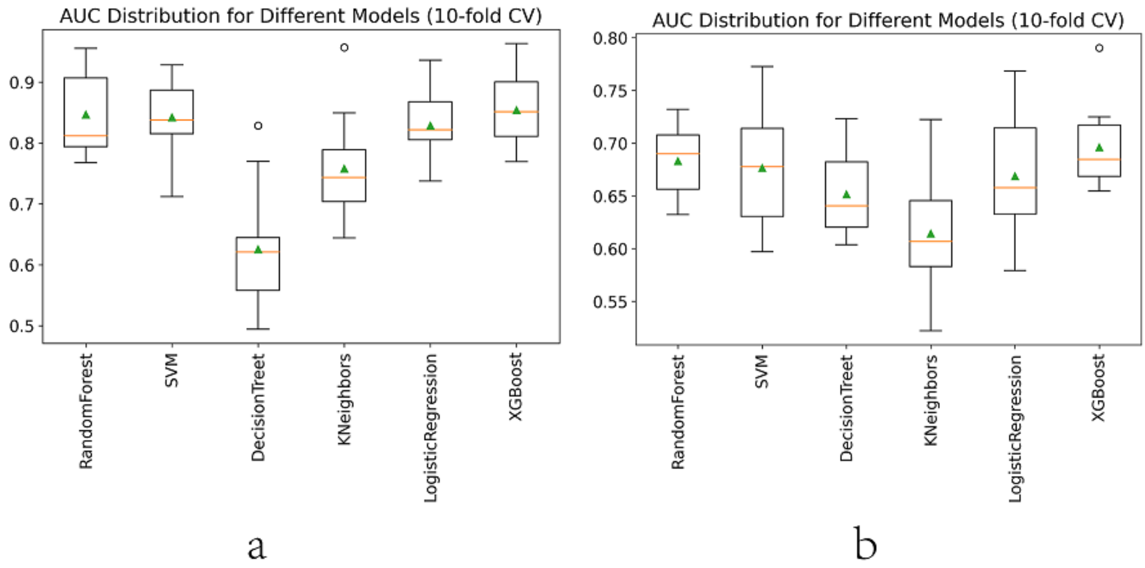

AUC, area under the curve; XGBoost, extreme gradient boosting; SVM, support vector machine.

Supplementary Fig. 8 SHAP summary bar plots.

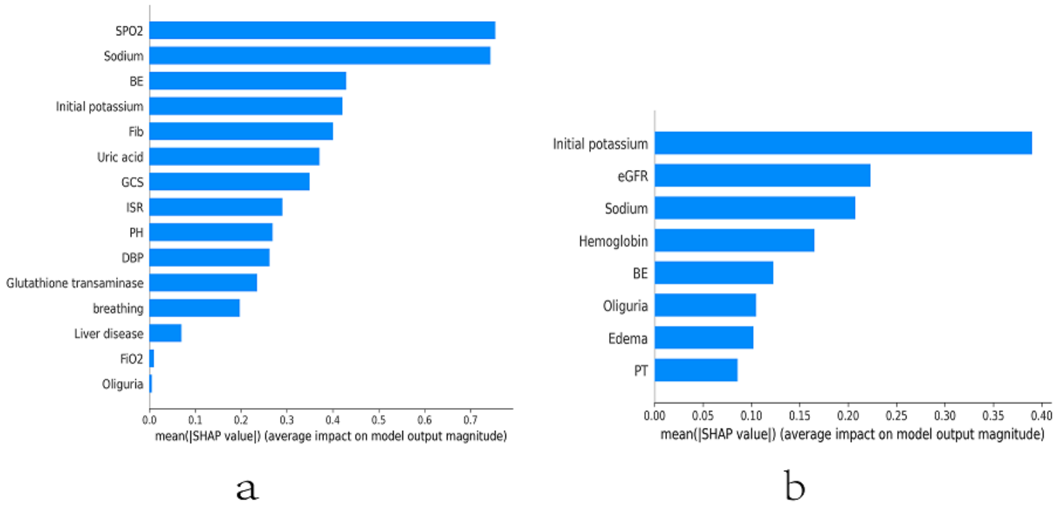

a, adverse outcomes; b, treatment effectiveness; Abbreviations: DBP, diastolic blood pressure; SpO_2, oxygen saturation; GCS, Glasgow coma score; ISR, International standardized ratio; eGFR, estimated glomerular filtration rate; BE, base excess; FiO_2, fraction of inspiration oxygen; Fib, fibrinogen; PT, prothrombin time.

Supplementary Table.1 Demographic and clinical characteristics at baseline.

		without adverse outcomes (n=981)	with adverse outcomes (n=93)	P value
**Age, year**		65.00(53,77)	71.00(54.00,83.00)	0.043
**Gender (male), n (%)**	1	632(64.424)	57(61.290)	0.625
**Temperature, ℃**		36.60(36.2,37.0)	36.40(35.90,37.00)	0.018
**Heart rate, bpm**		84.0(74.0,99.0)	86.00(69.00,109.00)	0.929
**SBP, mmHg**		136.0(115.0,157.0)	118.00(92.00,148.00)	<0.001
**DBP, mmHg**		75.0(63.0,88.0)	58.00(50.0,74.00)	<0.001
**MAP, mmHg**		96.0(83.0,109.0)	80.67(66.00,98.00)	<0.001
**Respiratory rate**		20.0(19.0,21.0)	20.00(20.00,24.00)	<0.001
**SpO_2, %**		99.0(97.0,100.0)	98.00(91.00,99.00)	<0.001
**FiO_2, mmHg**		21.0(21.0,21.0)	21.00(21.00,21.00)	<0.001
**GCS**		15.0(15.00, 15.00)	15.00(15.00, 15.00)	<0.001
**Edema, n (%)**	1	258(26.300)	41(44.086)	<0.001
**Smoking, n (%)**	1	247(25.178)	22(23.656)	0.843
**Drinking, n (%)**	1	186(18.960)	16(17.204)	0.783
**HTN, n (%)**	1	664(67.686)	54(58.065)	0.077
**DM, n (%)**	1	281(28.644)	31(33.333)	0.405
**Heart failure, n (%)**	1	140(14.271)	19(20.430)	0.148
**Liver disease, n (%)**	1	107(10.907)	25(26.882)	<0.001
**Tumors, n (%)**	1	177(18.043)	22(23.656)	0.233
**Chronic kidney disease, n (%)**	1	614(62.589)	49(52.688)	0.077
**Diabetic Nephropathy, n (%)**	1	74(7.543)	9(9.677)	0.594
**oliguria, n (%)**	1	173(17.635)	36(38.710)	<0.001
**High potassium history, n (%)**	1	73(7.441)	3(3.223)	0.192
**Acute gastrointestinal bleeding, n (%)**	1	82(8.359	16(17.204)	0.008
**Leukocyte count, 10^9/L**		8.20(6.00,11.9)	11.5(8.3,16.40)	<0.001
**Erythrocyte count, 10^12/L**		3.36(2.66,3.98)	3.1(2.31,3.99)	0.084
**Hemoglobin, g/L**		100(80.0,118.0)	94.00(68.00,116.00)	0.047
**Erythrocyte pressure product, n (%)**		31(24.9,36.5)	28.6 (21.70,36.60)	0.020
**Platelet count, 10^9/L**		188(136.0,256.0)	160.00(98.00,219.00)	<0.001
**Glutathione transaminase, U/L**		20.00(14,35.00)	50(19.00,166.00)	<0.001
**Creatinine, μmol /L**		347.0(175,700)	394(202,679.0)	0.546
**eGFR**		13.82(6.33,29.78)	11.88(6.77,23.47)	0.454
**Urea, mg/dL**		21.64(15.04,31)	25.74(19,45.94)	<0.001

**Uric acid, µmol/L**	451(357,558)	549.0(393.0,763.0)	<0.001
**Sodium, mmol/L**	138.0(135,141)	135.0(130.0,139.0)	<0.001
**Chlorine, mmol/L**	103.0(98,108)	94.00(101.00,105.00)	<0.001
**Total calcium, mmol/L**	2.16(2.0,2.32)	2.02(1.89,2.180)	<0.001
**Inorganic phosphorus, mmol/L**	1.62(1.29,2.06)	2.06(1.47,2.710)	<0.001
**ISR**	1.07(0.99,1.23)	1.25(1.08,1.69)	<0.001
**Fib, g/L**	3.67(2.82,4.75)	2.96(1.95,4.4)	<0.001
**APTT, second**	29.7(26.2,34.7)	34.7(27.90,48.70)	<0.001
**PT, second**	12.4(11.6,14.2)	14.6(12.8,19.3)	<0.001
**TT, second**	17.7(16.8,18.9)	18.4(17,19.70)	0.022
**pH**	7.37(7.311,7.41)	7.31(7.2,7.4)	<0.001
**pCO_2, mmHg**	34.6(29.2,39.9)	30.8(25.40,37.4)	0.001
**pO_2, mmHg**	96.9 (71,120)	97.5 (60.7,136.00)	0.435
**Bicarbonate concentration, mmol/L**	19.7(16,23.3)	16.3(11.30,20.9)	<0.001
**Base excess, mmol/L**	-4.5(-8.5, -1.1)	-9.2(-14.80, -3.80)	<0.001
**LDH, U/L**	253(203,340)	333(243.0,642.0)	<0.001
**HBDH, U/L**	216(165,280)	274(198.0,490.0)	<0.001
**CKMB, U/L**	18(13.00,28.00)	33.00(19.00,50.00)	<0.001
**Initial potassium, mmol/L**	6.41(6.18,6.76)	6.56(6.3,7)	0.003

SBP, systolic blood pressure; DBP, diastolic blood pressure; MAP, mean arterial pressure; SpO_2, oxygen saturation; FiO_2, fraction of inspiration oxygen; GCS, Glasgow coma score; HTN, hypertension; DM, diabetes mellitus; eGFR, estimated glomerular filtration rate; ISR, International standardized ratio; Fib, fibrinogen; APTT, activated partial thromboplastin time; PT, prothrombin time; TT, thrombin time; pCO_2, partial pressure of carbon dioxide; pO_2, partial pressure of oxygen; LDH, lactate dehydrogenase; HBDH, hydroxybutyrate dehydrogenase; CK-MB, creatine kinase-MB;0,no;1,yes.

Supplementary Table. 2 All predictor variables for patients with hyperkalemia in the training and testing data sets of adverse outcomes.

	Training Data Set (SMOTE)			Validating Data Set		
	without adverse outcomes (n=686)	with adverse outcomes (n=686)	P	without adverse outcomes (n=295)	with adverse outcomes (n=28)	P
DBP, mmHg	75 [63,88]	61[52,72]	<0.001	75 [63,87.5]	57.5[39.8, 112]	<0.001
breathing	20(19,21)	21(20,24)	<0.001	20(19.5,20)	20(20,24.2)	0.054
SPO2, %	99(98,100)	97(92,98)	<0.001	99(97,100)	98(85.5, 92)	0.009
GCS	15(15,15)	15(15,15)	<0.001	15 (15,15)	15(13,15)	<0.001
Liver disease, n%(yes)	73(10.641)	70(10.204)	0.860	34(11.525)	7(0.25)	0.080
oliguria, n (%)(yes)	124(18.076)	111(16.181)	0.390	49(16.61)	12(42.857)	0.002
Fib, g/L	3.8[2.9,4.8]	3.1 [2.1,4.2]	<0.001	3.57[2.745,4.68]	3.5[2.6,5.4]	0.710
Uric acid	447.5 [354.5,552]	538 [423.2,771.5]	<0.001	457[358.5,589.5]	530.5[374.2, 707.8]	0.184
Sodium, mmol/L	138[135,141]	135 [130,138]	<0.001	138 [134,141]	134.5[130.8,140]	0.093
ISR	1.065[0.99,1.22]	1.317 [1.11,1.73]	<0.001	1.1 [1,1.2]	1.3[1.1,1.7]	<0.001
PH	7.37[7.31,7.41]	7.292 [7.206,7.373]	<0.001	7.4 [7.3,7.4]	7.3[7.2, 7.4]	0.004
Base excess, mmol/L	-4.65[-8.6, -1.025]	-9.715 [-14.892, -5.178]	<0.001	-4.2 [-8, -1.1]	-7.3[-13.9, -3.8]	0.006
Initial potassium	6.415 [6.19,6.76]	6.603 [6.354,6.947]	<0.001	6.4[6.2,6.8]	6.5[6.3,7.3]	0.029
FiO2, mmHg	21[21,21]	21[21,21]	<0.001	21[21,21]	21[21,21]	<0.001
Glutathione transaminase, U/L	20[14,36]	38.5[19,103.2]	<0.001	20[15,34]	59.5 [21.8,182]	<0.001

Abbreviations: DBP, diastolic blood pressure; SpO_2, oxygen saturation; GCS, Glasgow coma score; ISR, International standardized ratio;.

Supplementary Table. 3 All predictor variables for patients with hyperkalemia in the training and testing data sets of treatment effectiveness.

	Training Data Set			Validating Data Set		
	Last blood potassium≤5.5 (n=484)	Last blood potassium>5.5 (n=267)	P	Last blood potassium≤5.5 (n=208)	Last blood potassium>5.5 (n=115)	P
Edema, n (%) (yes)	113(16.329)	92(24.084)	0.001	56(8.092)	38(9.948)	0.302
Oliguria, n(%)(yes)	73 [10.549]	74 [19.372]	<0.001	38 [5.491]	24[6.283]	0.674
eGFR	16.8 [7.4,34.5]	10.7 [6,22]	<0.001	17.335[5.897,31.665]	8.25[4.605, 23.825]	<0.001
Urea, mg/dL	20.4 [14.4, 29.4]	23.6 [17.4,33.8]	<0.001	20.22 [13.2,31.62]	27.45[18.83,39.31]	<0.001
Sodium, mmol/L	138[135,141]	137 [133,140]	0.002	139 [135,141]	138[133,141]	0.147
Base excess, mmol/L	-4 [-8.2, -0.5]	-5.5 [-9.9, -1.6]	0.001	-4.35 [-7.8, -1.2]	--7.6[-10.6, -4.4]	<0.001
Initial potassium, mmol/L	6.3[6.2,6.7]	6.5 [6.3,6.9]	<0.001	6.305[6.14,6.683]	6.64[6.3,7.145]	<0.001
Hemoglobin, g/L	103[82.75,121.25]	95[77,114]	0.001	99[78,119]	95[73.5,113.5]	0.348

eGFR, estimated glomerular filtration rate.

Supplementary Table. 4 Multiple logistic regression analysis of adverse events.

Variables	R	SE.	Z	*P*	OR	OR(95%CI)
DBP	-0.017	0.007	-2.66	*0.008*	0.983	0.97~0.995
breathing	0.080	0.027	2.941	*0.003*	1.083	1.027~1.142
SPO2	-0.042	0.013	-3.249	0.001	0.959	0.934 ~ 0.983
GCS	-0.240	0.067	-3.525	<0.001	0.790	0.693 ~0.901
Liver disease						
0					1	
1	0.784	0.333	2.354	0.019	2.189	1.140~ 4.205
Oliguria						
0					1	
1	1.165	0.274	4.253	<0.001	3.207	1.874~5.486
sodium	-0.043	0.019	-2.315	0.021	0.958	0.923~ 0.993
ISR	0.464	0.173	2.683	0.007	1.591	1.133~2.234
Initial potassium	0.642	0.218	2.994	0.003	1.900	1.239~2.912

R, regression coefficient; SE, Standard error; OR, odds ratio; CI, confidence interval; DBP, diastolic blood pressure; SPO2, oxygen saturation; GCS, Glasgow coma score; ISR, international standardized ratio; 0, no; 1, yes.

Supplementary Table. 5 Performances of the six machine learning models for predicting in-hospital adverse events.

	**XGBoost**	**LR**	**KNN**	**DT**	**SVM**	**RF**
**AUC**	0.870	0.844	0.685	0.699	0.848	0.779
**Accuracy**	0.848	0.796	0.814	0.770	0.827	0.780
**F1 scores**	0.424	0.377	0.25	0.315	0.417	0.297
**Sensitivity**	0.643	0.714	0.357	0.607	0.714	0.556
**Specificity**	0.316	0.256	0.192	0.213	0.294	0.206

AUC, area under the curve; XGBoost, extreme gradient boosting; LR, logistic regression; KNN, k-nearest neighbor; DT, decision tree; SVM, support vector machine; RF, random forest.

Supplementary Table. 6 For predicting treatment effects, the mean and standard deviation of 100 AUCs were run after removing the randomization seed.

	XGBoost	LR	KNN	DT	SVM	RF
**Mean AUC**	0.750	0.699	0.605	0.685	0.693	0.702
**Standard deviation**	1.11e-16	5.259e-03	0.000	1.11e-16	9.4e-05	1.402e-02

AUC, area under the curve; XGBoost, extreme gradient boosting; LR, logistic regression; KNN, k-nearest neighbor; DT, decision tree; SVM, support vector machine; RF, random forest.

Supplementary Table. 7 For predicting adverse events, the mean and standard deviation of 100 AUCs were run after removing the random seed.

	XGBoost	LR	KNN	DT	SVM	RF
**Mean AUC**	0.869	0.844	0.685	0.701	0.848	0.753
Standard deviation	0.000	1.8e-04	1.11e-16	1.313e-03	1.11e-16	1.469e-02

AUC, area under the curve; XGBoost, extreme gradient boosting; LR, logistic regression; KNN, k-nearest neighbor; DT, decision tree; SVM, support vector machine; RF, random forest.
